# Large Vessel Adventitial Vasculitis Characterizes Patients with Critical Lower Limb Ischemia with as Compared to without Human Immunodeficiency Virus Infection

**DOI:** 10.1371/journal.pone.0106205

**Published:** 2014-08-29

**Authors:** Martin Brand, Angela J. Woodiwiss, Frederic Michel, Simon Nayler, Martin G. Veller, Gavin R. Norton

**Affiliations:** 1 Department of Surgery, School of Medicine, University of the Witwatersrand, Johannesburg, South Africa; 2 Cardiovascular Pathophysiology and Genomics Research Unit, School of Physiology, University of the Witwatersrand, Johannesburg, South Africa; 3 Gritzman and Thatcher Inc., Faculty of Health Sciences, University of the Witwatersrand, Johannesburg, South Africa; Uniformed Services University, United States of America

## Abstract

**Objectives:**

Whether a human immunodeficiency virus (HIV)-associated vasculitis in-part accounts for occlusive large artery disease remains uncertain. We aimed to identify the histopathological features that characterize large vessel changes in HIV sero-positive as compared to sero-negative patients with critical lower limb ischemia (CLI).

**Materials and Methods:**

Femoral arteries obtained from 10 HIV positive and 10 HIV negative black African male patients admitted to a single vascular unit with CLI requiring above knee amputation were subjected to histopathological assessment. None of the HIV positive patients were receiving antiretroviral therapy.

**Results:**

As compared to HIV negative patients with CLI, HIV positive patients were younger (p<0.01) and had a lower prevalence of hypertension (10 vs 90%, p<0.005) and diabetes mellitus (0 vs 50%, p<0.05), but a similar proportion of patients previously or currently smoked (80 vs 60%). 90% of HIV positive patients, but no HIV negative patient had evidence of adventitial leukocytoclastic vasculitis of the vasa vasorum (p<0.0001). In addition, 70% of HIV positive, but no HIV negative patient had evidence of adventitial slit-like vessels. Whilst T-lymphocytes were noted in the adventitia in 80% of HIV positive patients, T-lymphocytes were noted only in the intima in HIV negative patients. The presence of femoral artery calcified multilayered fibro-atheroma was noted in 40% of HIV positive and 90% of HIV negative patients with CLI.

**Conclusions:**

An adventitial vasculitis which characterizes large artery changes in CLI in HIV-infected as compared to non-infected patients, may contribute toward HIV-associated occlusive large artery disease.

## Introduction

There is increasing evidence that human immunodeficiency virus (HIV) infection is a risk factor for occlusive arterial disease including myocardial infarction (MI) [1]–[3] and peripheral arterial disease (PAD) [4], [5]. A number of mechanisms may explain this relationship. Partly through antiretroviral therapy (protease inhibitors), patients with HIV infections may have an increased prevalence of conventional cardiovascular risk factors [6]–[8], the consequence being an enhanced degree of atherosclerosis [6]–[11]. However, vascular pathology in HIV may occur in the absence of risk factors [12] through arterial wall infections [13], [14] producing a vasculitis either through direct effects [14] or through co-infections [15]–[17]. Whether an HIV-associated vasculitis accounts in-part for occlusive arterial disease nevertheless remains uncertain.

Although a number of vasculitis subtypes have been described in HIV infected patients [8], the prevailing hypothesis is that HIV-associated occlusive arterial disease (MI and PAD) is largely attributed to premature atherosclerosis [7], [8]. Thus, cardiovascular risk prevention in HIV-infected patients has focused on targeting conventional cardiovascular risk factors [7], [8]. Nevertheless, there is some evidence that occlusive arterial disease in HIV-infected patients may be attributed to a vasculitis. In this regard, in the absence of atheroma, adventitial inflammation has been described in large vessels in a series of 16 HIV positive patients, most of whom had aneurysmal pathology, but 3 of whom also had occlusive arterial disease [18]; and in large vessels of 4 amputated limbs of HIV positive patients with critical lower limb ischemia (CLI) [19], [20]. However, without an HIV-sero-negative group with matched pathology available for comparison, adventitial vasculitis in patients with occlusive arterial disease [18]-[20] could be attributed to an epiphenomenon. Hence, whether a vasculitis characterizes HIV-positive as compared to negative patients with occlusive arterial disease is unknown. In the present study we therefore aimed to compare large artery histopathological characteristics in untreated HIV-sero-positive patients with CLI requiring amputation, to large artery characteristics in HIV-sero-negative patients with CLI requiring amputation.

## Materials and Methods

### Study groups and clinical data

The present study was conducted according to the principles outlined in the Helsinki declaration. The Committee for Research on Human Subjects of the University of the Witwatersrand approved the protocol (approval number: M120739). Participants gave informed, written consent. Ten HIV positive and 10 HIV negative black African male patients with CLI requiring above knee amputations were recruited from the Division of Vascular Surgery at the Chris Hani Baragwanath Hospital, Johannesburg, South Africa over the period September 2012 to July 2013. None of these patients had clinical evidence of aneurysms. All patients had ischemic pain at rest and evidence of tissue loss (necrosis as evidenced by foot and leg ulcers and/or gangrene). Routine HIV serology (ELISA) was performed to determine HIV status. A CD4 count was obtained in all HIV positive patients with CLI. Participants were considered to have diabetes mellitus if they had a fasting plasma glucose concentration ≥7 mmol/l, or in whom glucose-lowering agents were prescribed. Brachial blood pressure (BP) was measured according to guidelines and taken as the mean of five measurements. Participants with a BP ≥140/90 mm Hg or in those receiving antihypertensive medication were considered to have hypertension. Dyslipidemia was diagnosed as the presence of either a raised triglyceride concentration (≥1.7 mmol/l) or a reduced high density lipoprotein (HDL) concentration (<1.0 mmol/l).

#### Histopathological assessment

Specimens from the distal superficial femoral artery (SFA) were obtained at the time of above knee amputations due to PAD where reconstruction could not be performed resulting in end-stage CLI. One centimeter of the SFA was obtained at the level of transection for the amputation (from the viable stump). Specimens were fixed in 10% buffered formalin, processed according to standard methods and 4 µm sections cut and stained with hematoxylin and eosin. Sections were also stained with Elastic Von Giesson stain for elastic fibres; a Masson trichrome stain for collagen and an Alcian Blue stain for mucopolysaccharides. Immunohistochemical stains for CD3 (T-cell marker), CD20 (B-cell marker) and CD68 (histiocytic marker) were performed. The slides were assessed by a pathologist (SN) who was unaware of (blinded to) the HIV-status and clinical characteristics of the patients from whom the specimens were derived. The slides were evaluated for the presence of atheroma, fragmentation and reduplication of the internal elastic lamina, and adventitial vascular proliferation and perivascular inflammation. All parameters were graded semi-quantitatively, as 0 (absent), 1+(focally present), 2+(moderate) and 3+(extensive).

#### Data analysis

Continuous data are shown as mean ± SD. Proportions between groups were compared using a Fischer’s Exact test and continuous data were compared using a Mann-Whitney ranked sum test.

## Results

### Participant characteristics

The demographic and clinical characteristics of the participants are shown in Table 1. As compared to HIV negative patients with CLI, HIV positive patients with CLI were younger, and had a lower prevalence of traditional cardiovascular risk factors, including hypertension and diabetes mellitus (Table 1). HIV negative patients had higher glycated hemoglobin values (Table 1). A similar proportion of HIV positive patients with CLI smoked regularly as compared to HIV negative patients with CLI and similar average lipid concentrations were noted between the groups (Table 1). A wide range (74 to 643×10^6^/l) of values for CD4 count were obtained and 5 (50%) HIV positive participants had values that would not have qualified for antiretroviral therapy based on the current thresholds for therapy in South Africa (<350×10^6^/l), and 2 patients had CD4 counts >500×10^6^/l.

**Table 1:** Characteristics of black South African male patients with critical lower limb ischemia requiring above knee amputations with and without human immunodeficiency virus (HIV) infection recruited for the present study.

	HIV infected	HIV sero-negative
Sample size	10	10
Age (years)	47±12	62±6**
Previous cardiovascular event (n)	0	3
Body mass index (kg/m^2^)	22.4±6.1	25.3±3.9*
Diabetes mellitus (n)	0	5*
Glycated haemoglobin (%)	6.24±1.46	8.25±1.92*
Hypertension (n)	1	9**
Systolic blood pressure (mm Hg)	119±13	136±18*
Diastolic blood pressure (mm Hg)	79±12	78±10
Current smoking (n)	7	5
Previous smoking (n)	8	6
Total cholesterol (mmol/l)	3.11±1.11	3.95±1.53
LDL cholesterol (mmol/l)	1.86±0.97	2.61±1.26
HDL cholesterol (mmol/l)	0.62±0.32	0.80±0.33
CD4 count (×10^6^/l)	314±214	Not done

None of the HIV infected patients were receiving antiretroviral therapy.

LDL, low density lipoprotein concentrations; HDL, high density lipoprotein concentrations.

*p<0.05,

**p<0.01 vs HIV infected.

### Histopathological evidence of femoral wall inflammation

90% of HIV positive patients, but no HIV negative patient had evidence of adventitial leukocytoclastic vasculitis of the vasa vasorum (Table 2). In addition, 70% of HIV positive, but no HIV negative patient had evidence of adventitial slit-like vessels (Table 2). Whilst in HIV positive patients T-lymphocytes were noted in the adventitia in 80% and in the intima and media in 30% of patients, in HIV negative patients T-lymphocytes were noted only in the intima and these occurred in 50% of patients (Table 2). No B lymphocytes were observed in either group (data not shown). No significant differences in the mean (±SD) CD4 count were noted between HIV positive patients ranked as having 1+(366±218×10^6^/l) versus 2+(292±244×10^6^/l, p = 0.56) adventitial leukocytoclastic vasculitis.

**Table 2:** Inflammatory and associated changes in femoral artery sections obtained proximal to the occlusion in black South African male patients with critical lower limb ischemia requiring above knee amputations with (n = 10) and without (n = 10) human immunodeficiency virus (HIV) infection.

	HIV-infected		HIV-sero-negative	
	Number affected (n/10)	Severity†	Number affected (n/10)	Severity†
Adventitial changes				
Leukocytoclastic vasculitis	9/10**	1+, n = 5, 2+, n = 4	0/10	–
Slit like vessels	7/10*	1+, n = 5, 2+, n = 2	0/10	–
T-lymphocytes	7/10*	1+, n = 4, 2+, n = 3	0/10	–
Intimal or medial changes				
T-lymphocytes	3/10	1+, n = 3	5/10	1+, n = 4, 2+, n = 1

† Scoring system: 0 = absent, 1+ = focally present, 2+ = moderate.

*p<0.005,

**p = 0.0001 vs HIV sero-negative group.

### Histopathological evidence of arteriosclerotic changes

The presence of calcified multilayered fibro-atheroma was noted in 40% of HIV positive patients with CLI with none showing eccentric changes, whilst calcified multilayered fibro-atheroma was noted in 90% of HIV negative patients with CLI, 60% of whom had eccentric changes (Table 3). Ossification of plaque was noted in 30% of HIV negative patients with CLI, but no ossification was noted in HIV positive patients with CLI (Table 3). A similar proportion of HIV-positive and negative patients had evidence of macrophages and the extent of intracellular and extracellular lipid deposits was similar in both groups (Table 3). Lipid deposits were noted in HIV positive patients with multilayered fibro-atheroma only (Table 3). A similar proportion of HIV positive and HIV negative patients with CLI had fragmentation and reduplication of the internal elastic lamina and mucoid degeneration (Table 3). The combined thickness of the femoral artery intima and media tended (p = 0.20) to be lower in HIV positive as compared to negative patients with CLI (Table 3). However, intima-media thickness was similarly increased in both HIV positive (1.38±0.28, p<0.05) and HIV negative (1.56±0.79, p<0.05) patients with fibroatheroma as compared to HIV positive patients without fibroatheroma (0.74±0.41).

**Table 3:** Arteriosclerotic changes in femoral artery sections obtained proximal to the occlusion in black South African male patients with critical lower limb ischemia requiring above knee amputations with (n = 10) and without (n = 10) human immunodeficiency virus (HIV) infection.

	HIV-infected		HIV-sero-negative	
	Number	Severity†	Number	Severity†
	affected (n/10) or mean ± SD		affected (n/10) or mean ± SD	
Multi-layered fibro-atheroma	4/10	–	9/10	–
Calcification of plaque	4/10	–	9/10	–
Ossification of plaque	0/10	–	3/10	–
Eccentric plaque	0/10	–	6/10*	–
Lipid (intracellular)	4/10	1+, n = 0, 2+, n = 4	8/10	1+, n = 1, 2+, n = 6, 3+, n = 1
Lipid (extracellular)	4/10	1+, n = 0, 2+, n = 4	8/10	1+, n = 0, 2+, n = 6, 3+, n = 2
Fragmentation/reduplication of IEL	9/10	1+, n = 8, 2+, n = 1	10/10	1+, n = 6, 2+, n = 3, 3+, n = 1
Mucoid degeneration	9/10	1+, n = 5, 2+, n = 3, 3+, n = 1	9/10	1+, n = 5, 2+, n = 3, 3+, n = 1
Femoral intima+media thickness (mm)	1.00±0.48		1.45±0.82	

IEL, internal elastic lamina.

† Scoring system: 0 = absent, 1+ = focally present, 2+ = moderate, 3+ = extensive.

*p<0.05 vs HIV sero-negative group.

### Relationship between risk factors and advanced atheroma

All HIV positive patients with advanced atheroma were current or previous smokers (Table 4). However, HIV positive as compared to negative patients with advanced atheroma were younger and a trend (p = 0.05 and 0.11) for the presence of fewer HIV positive patients to have hypertension or diabetes mellitus was noted (Table 4).

**Table 4:** Risk factors in black South African male patients with critical lower limb ischemia requiring above knee amputations with (n = 4) and without (n = 9) human immunodeficiency virus (HIV) infection whom had evidence of advanced atheroma in the femoral arteries.

	HIV-infected	HIV-sero-negative
	Mean ± SD or %	Mean ± SD or %
Age (years)	45.3±9.9**	61.9±6.3
Hypertension	25%*	89%
Diabetes mellitus	0%	56%
Total cholesterol (mmol/l)	3.35±1.02	3.73±1.45
Smoking (current or past)	100%	67%

*p = 0.05,

**p<0.02 vs HIV sero-negative group.

## Discussion

The main findings of the present study are that as compared to femoral arteries from HIV negative African men with CLI, femoral arteries from HIV positive African men with CLI not receiving anti-retroviral therapy are largely characterized by the presence of leukocytoclastic vasculitis of the vasa vasorum and adventitial inflammation. In addition, a significant proportion of HIV positive men with CLI not receiving anti-retroviral therapy showed advanced femoral artery atheroma, despite a markedly lower age and a lower prevalence of conventional cardiovascular risk factors.

To the best of our knowledge the present study is the first to compare histopathological changes in appropriate large vessels in patients with and without HIV and with occlusive arterial disease. Indeed, although a number of vasculitis subtypes have been described in HIV infected patients [8], few studies have reported on large vessel changes in patients with occlusive large artery disease, such as MI, PAD or stroke. In this regard prior studies have reported on the presence of leukocytoclastic vasculitis of the vasa vasorum and adventitial inflammation in large vessels of patients with HIV and aneurysmal changes in which 3 patients had occlusive large artery disease [18], and in large vessels of 4 amputated limbs of HIV positive patients with critical limb ischemia (CLI) [19], [20]. In these studies [18]–[20], comparisons of histopathological features were not made with patients admitted for similar clinical events but without HIV. Hence, the histopathological changes reported on in HIV positive patients with occlusive arterial disease in these studies [18]–[20] may reflect an epiphenomenon and not HIV-associated pathology. However, the present study provides clear evidence that leukocytoclastic vasculitis of the vasa vasorum and adventitial inflammation in large vessels is a unique feature associated with HIV infection as compared to non-HIV-infection in patients with CLI.

Our finding of a characteristic adventitial inflammatory change in HIV positive as compared to negative patients with CLI in the present study should be interpreted with caution. These relationships may not represent cause and effect. A previous study reporting on leukocytoclastic vasculitis of the vasa vasorum and adventitial inflammation in patients with mainly large artery aneurysms and in a small number of patients with occlusive arterial disease (n = 3), also showed adventitial and medial fibrosis and loss of medial muscle [18]. The authors speculated that occlusion of the vasa vasorum leads to death of areas of large vessel walls and hence to aneurysm formation. However, in the present study we failed to note similar changes in any of the 10 patients with HIV and CLI. Nevertheless, to avoid the effects of ischemia on histopathological changes, we sampled femoral artery tissue proximal to the occlusion, rather than at the level of the occlusion. It is therefore possible that at the level of the occlusion, adventitial and medial fibrosis and loss of medial muscle may have been noted, the consequence being wall scarring, thrombus formation and ultimately vascular occlusion.

The present study provides intriguing evidence to suggest that at least in a portion of patients with HIV not receiving antiretroviral therapy who develop CLI (4 of 10 in the present study), advanced large artery atheroma (calcified fibroatheroma) may contribute toward vascular occlusion. In this regard, although HIV positive patients with CLI with advanced femoral artery were younger and tended to have less risk factors than HIV negative patients with CLI with advanced femoral artery atheroma, all HIV positive patients with advanced femoral artery atheroma were current or previous smokers. This is entirely consistent with a number of studies that have reported a high prevalence of smoking in HIV positive patients with cardiovascular disease [7], [8]. These data therefore suggest that conventional risk stratification may not apply equally or as effectively among those with as compared to those without HIV infections. Indeed, non-human primate models of immunodeficiency virus infections are associated with atherosclerotic lesions in the absence of conventional cardiovascular risk factors [21]. Large studies are required to evaluate whether current risk assessment charts adequately risk predict in HIV positive patients not receiving antiretroviral therapy in South Africa.

The findings that fewer HIV positive as compared to negative patients with CLI had advanced calcific fibroatheroma, and that femoral artery intima-media thickness tended to be lower in HIV positive as compared to negative patients, must also be interpreted with caution. As previously emphasized, to avoid the effects of ischemia on histopathological changes, we sampled femoral artery tissue proximal to the occlusion, rather than at the level of the occlusion. Hence, we may have missed areas of fibroatheroma in HIV positive patients and consequently, it is still possible that atheroma is the major cause of CLI in HIV positive patients not receiving antiretroviral therapy.

The clinical implications of the present study warrant consideration. In this regard, in two large vascular units in South Africa in which we have previously reported on very high admission rates for CLI, approximately 12% of these patients were HIV positive with low cardiovascular risk scores irrespective of whether or not they were receiving antiretroviral therapy [22]. In this regard, the present study suggests that the pathogenesis of CLI in these circumstances may involve a combination of a vasculitis and/or advanced atheroma formation, despite a low cardiovascular risk. These findings may have implications for both risk prevention, where a more aggressive approach to risk management is required in HIV positive patients who smoke, and for decisions regarding revascularization procedures, where the causal lesions may in-part be atheromatous in nature despite a lower overall cardiovascular risk. Moreover, the present study suggests that a significant proportion of HIV positive patients (50%) may develop CLI when they have a CD4 count above the threshold that qualifies for antiretroviral therapy in South Africa. If confirmed in larger studies, these data would suggest that specialized screening procedures, such as measures of carotid intima-media thickness [22] may be required in HIV positive patients with CD4 counts above the threshold for therapy, and that antiretrovirals are instituted before the CD4 count decreases to below the threshold for therapy in those patients considered at risk for CLI.

There are a number of limitations to the present study that warrant consideration. The small study sample raises the question of false positive and negative findings and hence a much larger study is required to confirm aspects of the present data. Nevertheless, it is unlikely that a larger study will improve on our ability to show differences in the presence of a leukocytoclastic vasculitis in 9 of 10 HIV positive patients and none of the 10 HIV negative patients. In addition, because CLI is associated with disturbances of coagulation and fibrinolytic systems before, during and up to 30 days after surgery [23] we were unable to assess the relationship between coagulation profiles and HIV in CLI. Further, as a consequence of limb ischemia and the presence of ulcers and/or gangrene (tissue necrosis) in all patients, which affects circulating inflammatory and immune activation markers [23], we were also unable to assess the relationship between inflammation or immune activation and HIV in CLI. Hence, we cannot exclude the possibility that a pro-coagulation state, well recognized to characterize HIV and which is associated with large artery changes in non-human primate models of immunodeficiency virus infections [21], is the main role player for CLI in HIV or whether inflammation and immune activation are important causes of HIV-associated CLI.

In conclusion, in the present study we show that as compared to HIV negative patients with CLI, leukocytoclastic vasculitis of the vasa vasorum and adventitial inflammation characterize large artery pathology in HIV positive patients with CLI not receiving anti-retroviral therapy. Moreover, we show that a significant proportion of HIV positive patients with CLI not receiving anti-retroviral therapy have advanced femoral artery atheromatous changes, despite a lower cardiovascular risk. These data may have implications for both primary prevention and for the management of occlusive arterial disease in HIV positive patients.

## Supporting Information

Table S1 **Database.**
(ZIP)
